# Bis(8-methyl-2,8-dicarba-*closo*-dodeca­boran-2-yl) tris­elenide

**DOI:** 10.1107/S1600536811025712

**Published:** 2011-07-06

**Authors:** Adriana Ilie, Albert Soran, Antonio Laguna, Cristian Silvestru

**Affiliations:** a Universitatea Babes-Bolyai, Facultatea de Chimie si Inginerie Chimica, Arany Janos No 11, 400028 Cluj-Napoca, Romania; b Inorganic Chemistry Department ICMA, University of Zaragoza-CSIC, 12 Pedro Cerbuna, 50009 Zaragoza, Spain

## Abstract

In the title compound, C_6_H_26_B_20_Se_3_, the geometry around the central Se atom is V-shaped, with the Se—Se—Se angle being 105.60 (4)°. The Se—Se bond lengths are consistent with single covalent bonds.

## Related literature

For general background to diorganotriselenides, see: Atanassov *et al.* (2004 ▶); Hansen *et al.* (1989 ▶); Klapötke *et al.* (2006 ▶, 2007 ▶, 2008 ▶); Kulcsar *et al.* (2007 ▶); Kumar *et al.* (2004 ▶).
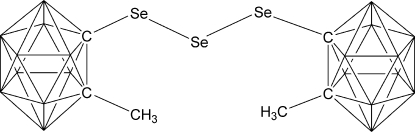


## Experimental

### 

#### Crystal data

C_6_H_26_B_20_Se_3_
*M* *_r_* = 551.35
Orthorhombic, 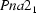

*a* = 18.947 (2) Å
*b* = 11.2734 (13) Å
*c* = 10.8533 (12) Å
*V* = 2318.2 (5) Å^3^
*Z* = 4
Mo *K*α radiation
μ = 4.75 mm^−1^
*T* = 297 K
0.40 × 0.30 × 0.20 mm

#### Data collection

Bruker SMART CCD area-detector diffractometer
Absorption correction: multi-scan (*SADABS*; Bruker, 2000 ▶) *T*_min_ = 0.252, *T*_max_ = 0.450
16082 measured reflections
4083 independent reflections
3398 reflections with *I* > 2σ(*I*)
*R*_int_ = 0.069

#### Refinement

*R*[*F* ^2^ > 2σ(*F* ^2^)] = 0.053
*wR*(*F* ^2^) = 0.113
*S* = 1.05
4083 reflections
264 parameters
1 restraint
H-atom parameters constrained
Δρ_max_ = 0.61 e Å^−3^
Δρ_min_ = −0.38 e Å^−3^
Absolute structure: Flack (1983 ▶), 1920 Friedel pairs
Flack parameter: 0.01 (2)

### 

Data collection: *SMART* (Bruker, 2000 ▶); cell refinement: *SAINT-Plus* (Bruker, 2001 ▶); data reduction: *SAINT-Plus*; program(s) used to solve structure: *SHELXS97* (Sheldrick, 2008 ▶); program(s) used to refine structure: *SHELXL97* (Sheldrick, 2008 ▶); molecular graphics: *DIAMOND* (Brandenburg, 2006 ▶); software used to prepare material for publication: *enCIFer* (Allen *et al.*, 2004 ▶) and *publCIF* (Westrip, 2010 ▶).

## Supplementary Material

Crystal structure: contains datablock(s) I, global. DOI: 10.1107/S1600536811025712/pk2331sup1.cif

Structure factors: contains datablock(s) I. DOI: 10.1107/S1600536811025712/pk2331Isup2.hkl

Additional supplementary materials:  crystallographic information; 3D view; checkCIF report

## Figures and Tables

**Fig. 1.**
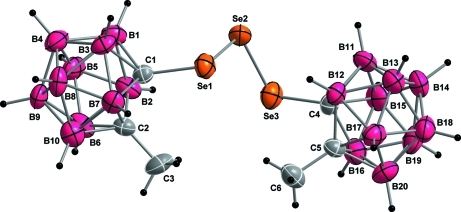
Graphical representation of the molecular structure of [(MeC2B10H10)2Se3]. Displacement ellipsoids are drawn at the 50% probability level.

## supplementary crystallographic information

### Comment

Several diorganotriselenides of type R—Se—Se—Se—*R* have been reported: *R* = 2,6-(2,4,6-Me_3_C_6_H_2_)_2_C_6_H_3_ and *R* = 2,6-(2,4,6 - *i*Pr_3_C_6_H_2_)_2_C_6_H_3_ (Klapötke *et. al.*, 2007); *R* = 2-(Me_2_NCH_2_)C_6_H_4_– (Kulcsar *et al.*, 2007); *R* = (Me_2_PhSi)_2_ClC (Klapötke *et al.*, 2007); *R* = (Me_2_PhSi)_3_C (Klapötke *et al.*, 2006); *R* = 4-(phenylamino) quinazolin-2-yl (Atanassov *et al.*, 2004); *R* = 2-(2-phenyl-5,6- dihydro-4*H*-1,3-oxazinyl) (Kumar *et al.*, 2004). We report here the structure of the title compound, the first (to our knowledge), dicarboranetriselenide described so far.

The asymmetric unit of the title compound (Fig. 1) consists of a discrete molecule.  The geometry around the central Se atom is V-shaped, with a Se—Se—Se angle of 105.60 (4)°. The Se—Se bond lengths are consistent with single covalent bonds.  The values for the Se—Se bond distances of 2.3102 (12) Å and 2.3120 (13) Å, as well as for the torsion angles C1—Se1—Se2—Se3 -95.3 (2)° and C4—Se3—Se2—Se1 -84.8 (2)°, compare well with the corresponding values found in other triselenides (Kulcsar *et al.*, 2007; Klapötke *et al.*, 2007; Klapötke *et al.*, 2006; Hansen *et al.*, 1989).

### Experimental

A mixture of [2-(MeC_2_B_10_H_10_SeCH_2_)py] (0.184 g, 5.6 mmol) and [Cu(MeCN)_4_]PF_6_ (0.208 g, 5.6 mmol) in CDCl_3_ was stirred for 2 h. The resulting solution was filtered and allowed to stand at room temperature. After two weeks, yellow crystals of the title compound [(MeC_2_B_10_H_10_)_2_Se_3_] were obtained.

### Refinement

All hydrogen atoms were placed in calculated positions using a riding model, with C—H = 0.96 Å and B—H = 1.1 Å with *U*_iso_=1.5Ueq (C) for methyl H and *U*_iso_=1.2Ueq (B) for the rest of H. The methyl groups were allowed to rotate while retaining tetrahedral geometry.

### Crystal data

**Table:**

C_6_H_26_B_20_Se_3_	*F*(000) = 1056
*M**_r_* = 551.35	*D*_x_ = 1.580 Mg m^−^^3^
Orthorhombic, *P**n**a*2_1_	Mo *K*α radiation, λ = 0.71073 Å
Hall symbol: P 2c -2n	Cell parameters from 2078 reflections
*a* = 18.947 (2) Å	θ = 2.6–18.5°
*b* = 11.2734 (13) Å	µ = 4.75 mm^−^^1^
*c* = 10.8533 (12) Å	*T* = 297 K
*V* = 2318.2 (5) Å^3^	Block, yellow
*Z* = 4	0.40 × 0.30 × 0.20 mm

### Data collection

**Table:**

Bruker SMART CCD area-detector diffractometer	4083 independent reflections
Radiation source: fine-focus sealed tube	3398 reflections with *I* > 2σ(*I*)
graphite	*R*_int_ = 0.069
φ and ω scans	θ_max_ = 25.0°, θ_min_ = 2.1°
Absorption correction: multi-scan (*SADABS*; Bruker, 2000)	*h* = −22→22
*T*_min_ = 0.252, *T*_max_ = 0.450	*k* = −13→13
16082 measured reflections	*l* = −12→12

### Refinement

**Table:**

Refinement on *F*^2^	Secondary atom site location: difference Fourier map
Least-squares matrix: full	Hydrogen site location: inferred from neighbouring sites
*R*[*F*^2^ > 2σ(*F*^2^)] = 0.053	H-atom parameters constrained
*wR*(*F*^2^) = 0.113	*w* = 1/[σ^2^(*F*_o_^2^) + (0.0497*P*)^2^] where *P* = (*F*_o_^2^ + 2*F*_c_^2^)/3
*S* = 1.05	(Δ/σ)_max_ = 0.001
4083 reflections	Δρ_max_ = 0.61 e Å^−^^3^
264 parameters	Δρ_min_ = −0.38 e Å^−^^3^
1 restraint	Absolute structure: Flack (1983), 1920 Friedel pairs
Primary atom site location: structure-invariant direct methods	Flack parameter: 0.01 (2)

### Special details

**Table:**

Geometry. All e.s.d.'s (except the e.s.d. in the dihedral angle between two l.s. planes) are estimated using the full covariance matrix. The cell e.s.d.'s are taken into account individually in the estimation of e.s.d.'s in distances, angles and torsion angles; correlations between e.s.d.'s in cell parameters are only used when they are defined by crystal symmetry. An approximate (isotropic) treatment of cell e.s.d.'s is used for estimating e.s.d.'s involving l.s. planes.
Refinement. Refinement of *F*^2^ against ALL reflections. The weighted *R*-factor *wR* and goodness of fit *S* are based on *F*^2^, conventional *R*-factors *R* are based on *F*, with *F* set to zero for negative *F*^2^. The threshold expression of *F*^2^ > σ(*F*^2^) is used only for calculating *R*-factors(gt) *etc*. and is not relevant to the choice of reflections for refinement. *R*-factors based on *F*^2^ are statistically about twice as large as those based on *F*, and *R*- factors based on ALL data will be even larger.

### Fractional atomic coordinates and isotropic or equivalent isotropic displacement parameters (Å^2^)

**Table:**

	*x*	*y*	*z*	*U*_iso_*/*U*_eq_	
B1	0.8152 (5)	−0.3444 (9)	0.1248 (8)	0.049 (2)	
H1	0.8670	−0.3770	0.0938	0.059*	
B2	0.8058 (4)	−0.2509 (8)	0.2541 (8)	0.042 (2)	
H2	0.8520	−0.2206	0.3068	0.051*	
B3	0.7474 (5)	−0.3072 (7)	0.0166 (9)	0.045 (2)	
H3	0.7554	−0.3158	−0.0834	0.055*	
B4	0.7359 (5)	−0.4328 (9)	0.1164 (9)	0.056 (3)	
H4	0.7359	−0.5246	0.0820	0.067*	
B5	0.7735 (6)	−0.3945 (7)	0.2635 (9)	0.053 (2)	
H5	0.7987	−0.4607	0.3236	0.064*	
B6	0.7228 (5)	−0.2797 (9)	0.3243 (9)	0.051 (2)	
H6	0.7145	−0.2702	0.4241	0.061*	
B7	0.6993 (4)	−0.1897 (8)	0.0820 (9)	0.044 (2)	
H7	0.6766	−0.1191	0.0244	0.052*	
B8	0.6658 (5)	−0.3341 (8)	0.0858 (10)	0.051 (2)	
H8	0.6203	−0.3608	0.0295	0.061*	
B9	0.6807 (5)	−0.3890 (8)	0.2379 (9)	0.052 (2)	
H9	0.6445	−0.4526	0.2816	0.062*	
B10	0.6567 (5)	−0.2416 (9)	0.2160 (8)	0.050 (2)	
H10	0.6050	−0.2070	0.2448	0.060*	
B11	1.0502 (5)	0.1868 (9)	0.0637 (9)	0.052 (3)	
H11	1.0780	0.1126	0.0193	0.063*	
B12	0.9589 (4)	0.2115 (7)	0.0395 (8)	0.040 (2)	
H12	0.9263	0.1528	−0.0181	0.048*	
B13	1.0214 (5)	0.3096 (9)	−0.0207 (10)	0.054 (3)	
H13	1.0303	0.3162	−0.1206	0.064*	
B14	1.0909 (5)	0.3232 (9)	0.0901 (10)	0.055 (2)	
H14	1.1458	0.3398	0.0620	0.067*	
B15	1.0710 (5)	0.2338 (9)	0.2168 (9)	0.052 (3)	
H15	1.1123	0.1906	0.2720	0.062*	
B16	0.9918 (5)	0.2851 (8)	0.2848 (9)	0.052 (3)	
H16	0.9804	0.2735	0.3834	0.063*	
B17	0.9455 (5)	0.3657 (8)	0.0479 (9)	0.050 (2)	
H17	0.9046	0.4099	−0.0074	0.060*	
B18	1.0262 (5)	0.4347 (9)	0.0783 (10)	0.059 (3)	
H18	1.0388	0.5237	0.0431	0.070*	
B19	1.0556 (6)	0.3843 (9)	0.2281 (10)	0.059 (3)	
H19	1.0870	0.4404	0.2904	0.071*	
B20	0.9641 (5)	0.4120 (8)	0.2017 (10)	0.052 (2)	
H20	0.9357	0.4852	0.2468	0.063*	
C1	0.7868 (3)	−0.2037 (6)	0.1094 (7)	0.0353 (16)	
C2	0.7330 (4)	−0.1659 (7)	0.2261 (6)	0.0407 (18)	
C3	0.7363 (5)	−0.0404 (7)	0.2731 (8)	0.060 (2)	
H3A	0.7809	−0.0271	0.3125	0.091*	
H3B	0.6989	−0.0276	0.3313	0.091*	
H3C	0.7311	0.0137	0.2053	0.091*	
C4	0.9916 (4)	0.1729 (6)	0.1812 (7)	0.0371 (17)	
C5	0.9286 (4)	0.2786 (6)	0.1715 (7)	0.0382 (17)	
C6	0.8535 (4)	0.2487 (8)	0.2121 (8)	0.059 (2)	
H6A	0.8493	0.1646	0.2238	0.089*	
H6B	0.8207	0.2741	0.1499	0.089*	
H6C	0.8432	0.2887	0.2881	0.089*	
Se1	0.83965 (4)	−0.08061 (7)	0.02743 (8)	0.0485 (2)	
Se2	0.95387 (4)	−0.10616 (7)	0.09702 (9)	0.0543 (2)	
Se3	0.96638 (4)	0.02322 (7)	0.26085 (7)	0.0528 (2)	

### Atomic displacement parameters (Å^2^)

**Table:**

	*U*^11^	*U*^22^	*U*^33^	*U*^12^	*U*^13^	*U*^23^
B1	0.050 (6)	0.047 (5)	0.050 (6)	0.014 (4)	0.003 (4)	0.004 (4)
B2	0.037 (5)	0.061 (6)	0.028 (4)	0.007 (4)	−0.003 (4)	0.005 (5)
B3	0.057 (6)	0.042 (5)	0.038 (5)	0.004 (4)	−0.009 (5)	−0.012 (4)
B4	0.071 (7)	0.041 (5)	0.056 (6)	0.000 (5)	0.003 (5)	−0.011 (5)
B5	0.075 (7)	0.034 (5)	0.050 (5)	0.004 (4)	0.009 (5)	0.008 (5)
B6	0.039 (5)	0.072 (7)	0.042 (5)	0.002 (5)	0.005 (4)	0.008 (5)
B7	0.036 (5)	0.044 (5)	0.050 (5)	0.004 (4)	−0.010 (4)	0.003 (4)
B8	0.053 (6)	0.042 (5)	0.058 (5)	−0.017 (4)	−0.012 (5)	0.013 (5)
B9	0.062 (6)	0.031 (5)	0.062 (6)	−0.012 (4)	0.006 (5)	0.001 (4)
B10	0.048 (6)	0.056 (6)	0.045 (5)	0.000 (5)	−0.006 (4)	0.009 (5)
B11	0.036 (5)	0.059 (6)	0.062 (7)	0.005 (5)	0.010 (4)	−0.008 (5)
B12	0.049 (6)	0.035 (5)	0.036 (5)	−0.002 (4)	−0.004 (4)	0.003 (4)
B13	0.045 (6)	0.057 (7)	0.058 (6)	−0.002 (5)	0.003 (5)	−0.001 (5)
B14	0.041 (5)	0.061 (6)	0.065 (6)	−0.013 (4)	0.007 (5)	0.005 (6)
B15	0.030 (5)	0.065 (7)	0.061 (7)	−0.007 (5)	−0.013 (4)	−0.006 (5)
B16	0.046 (6)	0.058 (6)	0.053 (7)	−0.004 (5)	−0.007 (5)	0.001 (5)
B17	0.062 (6)	0.039 (5)	0.050 (6)	0.006 (5)	0.004 (5)	0.005 (4)
B18	0.065 (7)	0.048 (6)	0.063 (6)	−0.016 (5)	0.003 (5)	0.001 (5)
B19	0.064 (7)	0.051 (6)	0.062 (7)	−0.019 (5)	−0.012 (5)	−0.008 (5)
B20	0.060 (7)	0.040 (6)	0.057 (6)	0.012 (4)	−0.007 (5)	−0.008 (5)
C1	0.037 (4)	0.036 (4)	0.033 (4)	0.002 (3)	−0.003 (3)	−0.003 (3)
C2	0.039 (4)	0.047 (5)	0.036 (4)	0.005 (3)	0.004 (3)	−0.005 (3)
C3	0.081 (6)	0.044 (5)	0.057 (5)	0.007 (4)	−0.002 (5)	−0.019 (4)
C4	0.037 (4)	0.032 (4)	0.043 (4)	0.001 (3)	−0.005 (3)	0.006 (3)
C5	0.039 (4)	0.043 (4)	0.032 (4)	0.004 (4)	0.005 (3)	0.003 (3)
C6	0.050 (5)	0.065 (6)	0.063 (6)	0.003 (4)	0.014 (4)	0.011 (5)
Se1	0.0563 (5)	0.0459 (4)	0.0434 (4)	−0.0062 (4)	0.0029 (4)	0.0088 (4)
Se2	0.0457 (5)	0.0423 (4)	0.0749 (6)	0.0014 (4)	0.0136 (5)	−0.0047 (5)
Se3	0.0621 (5)	0.0472 (5)	0.0491 (4)	−0.0042 (4)	−0.0055 (5)	0.0111 (4)

### Geometric parameters (Å, °)

**Table:**

B1—C1	1.683 (11)	B12—C5	1.719 (11)
B1—B2	1.763 (13)	B12—B13	1.746 (13)
B1—B3	1.790 (12)	B12—B17	1.759 (12)
B1—B5	1.791 (13)	B12—H12	1.1000
B1—B4	1.806 (14)	B13—B17	1.738 (13)
B1—H1	1.1000	B13—B18	1.776 (14)
B2—C1	1.696 (11)	B13—B14	1.790 (14)
B2—C2	1.708 (11)	B13—H13	1.1000
B2—B5	1.734 (12)	B14—B15	1.746 (14)
B2—B6	1.778 (12)	B14—B18	1.761 (15)
B2—H2	1.1000	B14—B19	1.779 (15)
B3—C1	1.713 (10)	B14—H14	1.1000
B3—B8	1.745 (13)	B15—C4	1.699 (11)
B3—B7	1.758 (12)	B15—B19	1.725 (14)
B3—B4	1.796 (13)	B15—B16	1.768 (13)
B3—H3	1.1000	B15—H15	1.1000
B4—B9	1.754 (14)	B16—C4	1.693 (12)
B4—B8	1.764 (14)	B16—C5	1.719 (12)
B4—B5	1.801 (14)	B16—B19	1.758 (14)
B4—H4	1.1000	B16—B20	1.771 (13)
B5—B6	1.741 (13)	B16—H16	1.1000
B5—B9	1.781 (14)	B17—C5	1.693 (11)
B5—H5	1.1000	B17—B18	1.746 (13)
B6—C2	1.678 (12)	B17—B20	1.783 (14)
B6—B9	1.741 (13)	B17—H17	1.1000
B6—B10	1.770 (13)	B18—B20	1.800 (15)
B6—H6	1.1000	B18—B19	1.811 (15)
B7—C1	1.693 (10)	B18—H18	1.1000
B7—C2	1.710 (11)	B19—B20	1.786 (14)
B7—B8	1.747 (12)	B19—H19	1.1000
B7—B10	1.762 (13)	B20—C5	1.680 (12)
B7—H7	1.1000	B20—H20	1.1000
B8—B10	1.764 (14)	C1—C2	1.682 (9)
B8—B9	1.785 (14)	C1—Se1	1.928 (7)
B8—H8	1.1000	C2—C3	1.506 (11)
B9—B10	1.739 (13)	C3—H3A	0.9600
B9—H9	1.1000	C3—H3B	0.9600
B10—C2	1.681 (12)	C3—H3C	0.9600
B10—H10	1.1000	C4—C5	1.690 (10)
B11—C4	1.699 (12)	C4—Se3	1.955 (7)
B11—B14	1.743 (13)	C5—C6	1.527 (10)
B11—B13	1.747 (14)	C6—H6A	0.9600
B11—B12	1.772 (12)	C6—H6B	0.9600
B11—B15	1.789 (14)	C6—H6C	0.9600
B11—H11	1.1000	Se1—Se2	2.3102 (12)
B12—C4	1.715 (11)	Se2—Se3	2.3120 (13)
			
C1—B1—B2	58.9 (5)	B11—B13—B14	59.1 (5)
C1—B1—B3	59.0 (5)	B18—B13—B14	59.2 (6)
B2—B1—B3	108.0 (6)	B17—B13—H13	121.6
C1—B1—B5	103.9 (6)	B12—B13—H13	121.1
B2—B1—B5	58.4 (5)	B11—B13—H13	121.5
B3—B1—B5	108.0 (7)	B18—B13—H13	122.3
C1—B1—B4	104.5 (6)	B14—B13—H13	122.9
B2—B1—B4	106.7 (6)	B11—B14—B15	61.7 (5)
B3—B1—B4	59.9 (5)	B11—B14—B18	108.1 (6)
B5—B1—B4	60.1 (5)	B15—B14—B18	108.6 (7)
C1—B1—H1	124.7	B11—B14—B19	108.3 (7)
B2—B1—H1	122.3	B15—B14—B19	58.6 (6)
B3—B1—H1	121.1	B18—B14—B19	61.5 (6)
B5—B1—H1	123.0	B11—B14—B13	59.2 (5)
B4—B1—H1	122.7	B15—B14—B13	108.7 (6)
C1—B2—C2	59.2 (4)	B18—B14—B13	60.0 (5)
C1—B2—B5	105.8 (6)	B19—B14—B13	108.9 (7)
C2—B2—B5	104.4 (6)	B11—B14—H14	121.5
C1—B2—B1	58.2 (5)	B15—B14—H14	121.4
C2—B2—B1	106.0 (6)	B18—B14—H14	121.1
B5—B2—B1	61.6 (5)	B19—B14—H14	121.5
C1—B2—B6	105.4 (6)	B13—B14—H14	121.6
C2—B2—B6	57.5 (5)	C4—B15—B19	105.4 (7)
B5—B2—B6	59.4 (5)	C4—B15—B14	104.2 (6)
B1—B2—B6	108.7 (7)	B19—B15—B14	61.6 (6)
C1—B2—H2	123.6	C4—B15—B16	58.4 (5)
C2—B2—H2	124.2	B19—B15—B16	60.4 (6)
B5—B2—H2	122.7	B14—B15—B16	108.8 (7)
B1—B2—H2	121.3	C4—B15—B11	58.2 (5)
B6—B2—H2	122.5	B19—B15—B11	108.7 (7)
C1—B3—B8	104.6 (6)	B14—B15—B11	59.1 (6)
C1—B3—B7	58.4 (4)	B16—B15—B11	107.3 (6)
B8—B3—B7	59.8 (5)	C4—B15—H15	125.1
C1—B3—B1	57.4 (5)	B19—B15—H15	121.1
B8—B3—B1	108.2 (7)	B14—B15—H15	122.1
B7—B3—B1	106.5 (6)	B16—B15—H15	121.5
C1—B3—B4	103.6 (6)	B11—B15—H15	122.1
B8—B3—B4	59.7 (5)	C4—B16—C5	59.4 (4)
B7—B3—B4	106.7 (6)	C4—B16—B19	104.2 (7)
B1—B3—B4	60.5 (5)	C5—B16—B19	104.9 (7)
C1—B3—H3	125.5	C4—B16—B15	58.7 (5)
B8—B3—H3	122.1	C5—B16—B15	106.2 (6)
B7—B3—H3	122.4	B19—B16—B15	58.6 (5)
B1—B3—H3	121.9	C4—B16—B20	105.3 (6)
B4—B3—H3	122.8	C5—B16—B20	57.5 (5)
B9—B4—B8	61.0 (6)	B19—B16—B20	60.8 (6)
B9—B4—B3	107.7 (6)	B15—B16—B20	107.7 (7)
B8—B4—B3	58.7 (5)	C4—B16—H16	123.8
B9—B4—B5	60.1 (6)	C5—B16—H16	123.6
B8—B4—B5	108.2 (7)	B19—B16—H16	123.5
B3—B4—B5	107.3 (6)	B15—B16—H16	122.3
B9—B4—B1	107.6 (7)	B20—B16—H16	122.2
B8—B4—B1	106.7 (7)	C5—B17—B13	106.6 (7)
B3—B4—B1	59.6 (5)	C5—B17—B18	106.0 (7)
B5—B4—B1	59.5 (5)	B13—B17—B18	61.3 (6)
B9—B4—H4	121.3	C5—B17—B12	59.7 (5)
B8—B4—H4	122.0	B13—B17—B12	59.9 (5)
B3—B4—H4	122.5	B18—B17—B12	108.9 (7)
B5—B4—H4	121.8	C5—B17—B20	57.7 (5)
B1—B4—H4	122.5	B13—B17—B20	110.2 (7)
B2—B5—B6	61.5 (5)	B18—B17—B20	61.3 (6)
B2—B5—B9	107.9 (6)	B12—B17—B20	108.1 (7)
B6—B5—B9	59.2 (5)	C5—B17—H17	124.2
B2—B5—B1	60.0 (5)	B13—B17—H17	120.9
B6—B5—B1	109.1 (6)	B18—B17—H17	121.1
B9—B5—B1	107.1 (7)	B12—B17—H17	121.4
B2—B5—B4	108.2 (7)	B20—B17—H17	121.1
B6—B5—B4	107.2 (7)	B17—B18—B14	107.7 (7)
B9—B5—B4	58.7 (6)	B17—B18—B13	59.1 (5)
B1—B5—B4	60.4 (6)	B14—B18—B13	60.8 (6)
B2—B5—H5	121.0	B17—B18—B20	60.4 (6)
B6—B5—H5	121.2	B14—B18—B20	107.4 (7)
B9—B5—H5	122.9	B13—B18—B20	107.7 (7)
B1—B5—H5	121.4	B17—B18—B19	107.4 (7)
B4—B5—H5	122.3	B14—B18—B19	59.7 (6)
C2—B6—B9	104.6 (7)	B13—B18—B19	108.0 (7)
C2—B6—B5	105.3 (6)	B20—B18—B19	59.3 (6)
B9—B6—B5	61.5 (6)	B17—B18—H18	122.1
C2—B6—B10	58.3 (5)	B14—B18—H18	121.7
B9—B6—B10	59.4 (6)	B13—B18—H18	121.7
B5—B6—B10	108.6 (7)	B20—B18—H18	122.0
C2—B6—B2	59.1 (5)	B19—B18—H18	122.1
B9—B6—B2	107.7 (7)	B15—B19—B16	61.0 (6)
B5—B6—B2	59.0 (5)	B15—B19—B14	59.8 (6)
B10—B6—B2	107.3 (6)	B16—B19—B14	107.9 (7)
C2—B6—H6	124.6	B15—B19—B20	108.9 (7)
B9—B6—H6	122.2	B16—B19—B20	60.0 (5)
B5—B6—H6	121.6	B14—B19—B20	107.3 (7)
B10—B6—H6	121.9	B15—B19—B18	107.3 (7)
B2—B6—H6	122.1	B16—B19—B18	107.6 (7)
C1—B7—C2	59.2 (4)	B14—B19—B18	58.7 (6)
C1—B7—B8	105.3 (6)	B20—B19—B18	60.1 (6)
C2—B7—B8	105.1 (6)	B15—B19—H19	121.2
C1—B7—B3	59.5 (4)	B16—B19—H19	121.5
C2—B7—B3	107.1 (6)	B14—B19—H19	122.5
B8—B7—B3	59.7 (5)	B20—B19—H19	121.5
C1—B7—B10	105.8 (6)	B18—B19—H19	122.5
C2—B7—B10	57.9 (5)	C5—B20—B16	59.7 (5)
B8—B7—B10	60.4 (6)	C5—B20—B17	58.4 (5)
B3—B7—B10	108.7 (7)	B16—B20—B17	107.4 (6)
C1—B7—H7	123.3	C5—B20—B19	105.3 (6)
C2—B7—H7	123.5	B16—B20—B19	59.2 (5)
B8—B7—H7	123.1	B17—B20—B19	106.9 (7)
B3—B7—H7	121.2	C5—B20—B18	104.1 (6)
B10—B7—H7	122.1	B16—B20—B18	107.5 (7)
B3—B8—B7	60.5 (5)	B17—B20—B18	58.3 (6)
B3—B8—B4	61.6 (5)	B19—B20—B18	60.7 (6)
B7—B8—B4	108.6 (6)	C5—B20—H20	124.2
B3—B8—B10	109.2 (6)	B16—B20—H20	121.6
B7—B8—B10	60.3 (5)	B17—B20—H20	122.7
B4—B8—B10	107.2 (7)	B19—B20—H20	122.3
B3—B8—B9	108.5 (7)	B18—B20—H20	122.9
B7—B8—B9	106.7 (7)	C2—C1—B1	111.0 (6)
B4—B8—B9	59.2 (5)	C2—C1—B7	60.9 (5)
B10—B8—B9	58.7 (5)	B1—C1—B7	114.7 (6)
B3—B8—H8	120.2	C2—C1—B2	60.7 (4)
B7—B8—H8	121.8	B1—C1—B2	62.9 (5)
B4—B8—H8	121.5	B7—C1—B2	113.5 (6)
B10—B8—H8	122.0	C2—C1—B3	110.5 (5)
B9—B8—H8	122.9	B1—C1—B3	63.6 (5)
B10—B9—B6	61.1 (5)	B7—C1—B3	62.1 (5)
B10—B9—B4	108.8 (7)	B2—C1—B3	115.0 (6)
B6—B9—B4	109.3 (7)	C2—C1—Se1	118.7 (5)
B10—B9—B5	108.2 (6)	B1—C1—Se1	123.9 (5)
B6—B9—B5	59.3 (5)	B7—C1—Se1	111.1 (5)
B4—B9—B5	61.3 (6)	B2—C1—Se1	122.9 (5)
B10—B9—B8	60.1 (5)	B3—C1—Se1	116.4 (5)
B6—B9—B8	109.0 (6)	C3—C2—B6	120.6 (7)
B4—B9—B8	59.8 (6)	C3—C2—B10	122.3 (6)
B5—B9—B8	108.2 (7)	B6—C2—B10	63.6 (5)
B10—B9—H9	121.2	C3—C2—C1	117.9 (6)
B6—B9—H9	121.0	B6—C2—C1	110.7 (6)
B4—B9—H9	120.8	B10—C2—C1	110.1 (6)
B5—B9—H9	121.8	C3—C2—B2	115.7 (6)
B8—B9—H9	121.7	B6—C2—B2	63.3 (5)
C2—B10—B9	104.5 (7)	B10—C2—B2	114.9 (6)
C2—B10—B7	59.5 (5)	C1—C2—B2	60.1 (4)
B9—B10—B7	108.1 (7)	C3—C2—B7	118.2 (6)
C2—B10—B8	105.6 (6)	B6—C2—B7	114.7 (6)
B9—B10—B8	61.3 (5)	B10—C2—B7	62.6 (5)
B7—B10—B8	59.4 (5)	C1—C2—B7	59.9 (4)
C2—B10—B6	58.1 (5)	B2—C2—B7	112.1 (6)
B9—B10—B6	59.5 (6)	C2—C3—H3A	109.5
B7—B10—B6	107.7 (6)	C2—C3—H3B	109.5
B8—B10—B6	108.6 (7)	H3A—C3—H3B	109.5
C2—B10—H10	124.5	C2—C3—H3C	109.5
B9—B10—H10	122.2	H3A—C3—H3C	109.5
B7—B10—H10	121.6	H3B—C3—H3C	109.5
B8—B10—H10	121.6	C5—C4—B16	61.1 (5)
B6—B10—H10	121.9	C5—C4—B15	110.8 (6)
C4—B11—B14	104.3 (7)	B16—C4—B15	62.8 (5)
C4—B11—B13	105.2 (6)	C5—C4—B11	110.5 (6)
B14—B11—B13	61.7 (6)	B16—C4—B11	115.3 (6)
C4—B11—B12	59.2 (5)	B15—C4—B11	63.5 (5)
B14—B11—B12	108.5 (7)	C5—C4—B12	60.6 (4)
B13—B11—B12	59.5 (5)	B16—C4—B12	114.0 (6)
C4—B11—B15	58.2 (5)	B15—C4—B12	114.9 (6)
B14—B11—B15	59.2 (6)	B11—C4—B12	62.5 (5)
B13—B11—B15	108.8 (7)	C5—C4—Se3	117.6 (4)
B12—B11—B15	107.8 (6)	B16—C4—Se3	110.6 (5)
C4—B11—H11	124.9	B15—C4—Se3	117.7 (5)
B14—B11—H11	122.1	B11—C4—Se3	124.9 (5)
B13—B11—H11	121.5	B12—C4—Se3	121.9 (5)
B12—B11—H11	121.5	C6—C5—B20	120.9 (6)
B15—B11—H11	121.8	C6—C5—C4	119.0 (6)
C4—B12—C5	59.0 (4)	B20—C5—C4	109.6 (6)
C4—B12—B13	104.6 (6)	C6—C5—B17	122.2 (7)
C5—B12—B13	105.1 (6)	B20—C5—B17	63.8 (5)
C4—B12—B17	104.8 (6)	C4—C5—B17	108.9 (5)
C5—B12—B17	58.2 (5)	C6—C5—B16	116.9 (6)
B13—B12—B17	59.4 (5)	B20—C5—B16	62.8 (5)
C4—B12—B11	58.3 (5)	C4—C5—B16	59.5 (4)
C5—B12—B11	105.8 (6)	B17—C5—B16	114.1 (6)
B13—B12—B11	59.5 (5)	C6—C5—B12	117.1 (6)
B17—B12—B11	106.7 (6)	B20—C5—B12	115.0 (6)
C4—B12—H12	124.0	C4—C5—B12	60.4 (4)
C5—B12—H12	123.4	B17—C5—B12	62.1 (5)
B13—B12—H12	123.3	B16—C5—B12	112.5 (6)
B17—B12—H12	123.0	C5—C6—H6A	109.5
B11—B12—H12	122.5	C5—C6—H6B	109.5
B17—B13—B12	60.6 (5)	H6A—C6—H6B	109.5
B17—B13—B11	108.8 (7)	C5—C6—H6C	109.5
B12—B13—B11	61.0 (5)	H6A—C6—H6C	109.5
B17—B13—B18	59.6 (6)	H6B—C6—H6C	109.5
B12—B13—B18	108.1 (7)	C1—Se1—Se2	104.2 (2)
B11—B13—B18	107.2 (7)	Se1—Se2—Se3	105.60 (4)
B17—B13—B14	106.8 (7)	C4—Se3—Se2	103.3 (2)
B12—B13—B14	107.6 (7)		
			
B3—B1—B2—C1	−34.0 (5)	B11—B13—B18—B14	35.7 (6)
B5—B1—B2—C1	−134.4 (6)	B17—B13—B18—B20	37.3 (7)
B4—B1—B2—C1	−97.1 (6)	B12—B13—B18—B20	−0.4 (9)
C1—B1—B2—C2	36.4 (5)	B11—B13—B18—B20	−64.7 (8)
B3—B1—B2—C2	2.4 (8)	B14—B13—B18—B20	−100.5 (8)
B5—B1—B2—C2	−98.0 (7)	B17—B13—B18—B19	99.9 (7)
B4—B1—B2—C2	−60.7 (7)	B12—B13—B18—B19	62.2 (8)
C1—B1—B2—B5	134.4 (6)	B11—B13—B18—B19	−2.1 (9)
B3—B1—B2—B5	100.4 (7)	B14—B13—B18—B19	−37.9 (7)
B4—B1—B2—B5	37.3 (6)	C4—B15—B19—B16	38.7 (6)
C1—B1—B2—B6	96.9 (6)	B14—B15—B19—B16	136.8 (7)
B3—B1—B2—B6	62.9 (8)	B11—B15—B19—B16	99.8 (7)
B5—B1—B2—B6	−37.5 (6)	C4—B15—B19—B14	−98.1 (7)
B4—B1—B2—B6	−0.2 (8)	B16—B15—B19—B14	−136.8 (7)
B2—B1—B3—C1	34.0 (5)	B11—B15—B19—B14	−37.0 (6)
B5—B1—B3—C1	95.7 (7)	C4—B15—B19—B20	1.3 (9)
B4—B1—B3—C1	133.2 (6)	B14—B15—B19—B20	99.4 (8)
C1—B1—B3—B8	−96.0 (7)	B16—B15—B19—B20	−37.4 (7)
B2—B1—B3—B8	−62.0 (8)	B11—B15—B19—B20	62.4 (9)
B5—B1—B3—B8	−0.3 (8)	C4—B15—B19—B18	−62.2 (8)
B4—B1—B3—B8	37.2 (6)	B14—B15—B19—B18	35.9 (6)
C1—B1—B3—B7	−33.0 (6)	B16—B15—B19—B18	−100.9 (7)
B2—B1—B3—B7	1.0 (8)	B11—B15—B19—B18	−1.1 (9)
B5—B1—B3—B7	62.7 (8)	C4—B16—B19—B15	−38.6 (6)
B4—B1—B3—B7	100.2 (7)	C5—B16—B19—B15	−100.1 (7)
C1—B1—B3—B4	−133.2 (6)	B20—B16—B19—B15	−138.5 (7)
B2—B1—B3—B4	−99.2 (7)	C4—B16—B19—B14	−0.1 (9)
B5—B1—B3—B4	−37.5 (6)	C5—B16—B19—B14	−61.7 (8)
C1—B3—B4—B9	61.2 (8)	B15—B16—B19—B14	38.4 (6)
B8—B3—B4—B9	−37.9 (7)	B20—B16—B19—B14	−100.0 (8)
B7—B3—B4—B9	0.6 (8)	C4—B16—B19—B20	99.9 (7)
B1—B3—B4—B9	100.4 (8)	C5—B16—B19—B20	38.3 (6)
C1—B3—B4—B8	99.1 (7)	B15—B16—B19—B20	138.5 (7)
B7—B3—B4—B8	38.5 (6)	C4—B16—B19—B18	61.8 (8)
B1—B3—B4—B8	138.3 (7)	C5—B16—B19—B18	0.3 (9)
C1—B3—B4—B5	−2.1 (8)	B15—B16—B19—B18	100.4 (8)
B8—B3—B4—B5	−101.2 (7)	B20—B16—B19—B18	−38.0 (7)
B7—B3—B4—B5	−62.7 (8)	B11—B14—B19—B15	38.0 (6)
B1—B3—B4—B5	37.1 (6)	B18—B14—B19—B15	139.1 (7)
C1—B3—B4—B1	−39.2 (5)	B13—B14—B19—B15	100.9 (7)
B8—B3—B4—B1	−138.3 (7)	B11—B14—B19—B16	−1.0 (9)
B7—B3—B4—B1	−99.8 (7)	B15—B14—B19—B16	−39.0 (6)
C1—B1—B4—B9	−60.4 (8)	B18—B14—B19—B16	100.1 (7)
B2—B1—B4—B9	1.0 (9)	B13—B14—B19—B16	61.9 (9)
B3—B1—B4—B9	−100.6 (7)	B11—B14—B19—B20	−64.2 (9)
B5—B1—B4—B9	37.5 (6)	B15—B14—B19—B20	−102.2 (7)
C1—B1—B4—B8	3.8 (8)	B18—B14—B19—B20	36.9 (6)
B2—B1—B4—B8	65.1 (8)	B13—B14—B19—B20	−1.3 (9)
B3—B1—B4—B8	−36.4 (6)	B11—B14—B19—B18	−101.1 (7)
B5—B1—B4—B8	101.7 (7)	B15—B14—B19—B18	−139.1 (7)
C1—B1—B4—B3	40.2 (6)	B13—B14—B19—B18	−38.2 (6)
B2—B1—B4—B3	101.5 (7)	B17—B18—B19—B15	64.4 (9)
B5—B1—B4—B3	138.1 (7)	B14—B18—B19—B15	−36.3 (6)
C1—B1—B4—B5	−97.9 (7)	B13—B18—B19—B15	2.0 (9)
B2—B1—B4—B5	−36.6 (6)	B20—B18—B19—B15	102.3 (7)
B3—B1—B4—B5	−138.1 (7)	B17—B18—B19—B16	0.1 (9)
C1—B2—B5—B6	−98.9 (6)	B14—B18—B19—B16	−100.6 (7)
C2—B2—B5—B6	−37.3 (6)	B13—B18—B19—B16	−62.3 (9)
B1—B2—B5—B6	−138.0 (7)	B20—B18—B19—B16	38.0 (6)
C1—B2—B5—B9	−60.7 (8)	B17—B18—B19—B14	100.7 (7)
C2—B2—B5—B9	0.8 (8)	B13—B18—B19—B14	38.3 (6)
B1—B2—B5—B9	−99.8 (7)	B20—B18—B19—B14	138.6 (7)
B6—B2—B5—B9	38.2 (6)	B17—B18—B19—B20	−37.9 (6)
C1—B2—B5—B1	39.1 (6)	B14—B18—B19—B20	−138.6 (7)
C2—B2—B5—B1	100.6 (6)	B13—B18—B19—B20	−100.2 (7)
B6—B2—B5—B1	138.0 (7)	C4—B16—B20—C5	36.8 (5)
C1—B2—B5—B4	1.3 (8)	B19—B16—B20—C5	134.7 (7)
C2—B2—B5—B4	62.8 (8)	B15—B16—B20—C5	98.3 (7)
B1—B2—B5—B4	−37.8 (6)	C4—B16—B20—B17	1.8 (9)
B6—B2—B5—B4	100.2 (8)	C5—B16—B20—B17	−35.1 (6)
C1—B1—B5—B2	−39.0 (6)	B19—B16—B20—B17	99.7 (7)
B3—B1—B5—B2	−100.5 (7)	B15—B16—B20—B17	63.2 (8)
B4—B1—B5—B2	−137.9 (7)	C4—B16—B20—B19	−97.9 (7)
C1—B1—B5—B6	−0.5 (9)	C5—B16—B20—B19	−134.7 (7)
B2—B1—B5—B6	38.5 (6)	B15—B16—B20—B19	−36.4 (6)
B3—B1—B5—B6	−62.0 (8)	C4—B16—B20—B18	−59.7 (8)
B4—B1—B5—B6	−99.4 (8)	C5—B16—B20—B18	−96.5 (7)
C1—B1—B5—B9	62.1 (8)	B19—B16—B20—B18	38.3 (7)
B2—B1—B5—B9	101.2 (7)	B15—B16—B20—B18	1.8 (9)
B3—B1—B5—B9	0.6 (8)	B13—B17—B20—C5	−97.4 (7)
B4—B1—B5—B9	−36.8 (6)	B18—B17—B20—C5	−135.7 (7)
C1—B1—B5—B4	98.9 (7)	B12—B17—B20—C5	−33.6 (6)
B2—B1—B5—B4	137.9 (7)	C5—B17—B20—B16	35.6 (6)
B3—B1—B5—B4	37.4 (6)	B13—B17—B20—B16	−61.8 (9)
B9—B4—B5—B2	−100.3 (7)	B18—B17—B20—B16	−100.1 (7)
B8—B4—B5—B2	−61.4 (9)	B12—B17—B20—B16	2.0 (9)
B3—B4—B5—B2	0.5 (9)	C5—B17—B20—B19	97.9 (7)
B1—B4—B5—B2	37.7 (6)	B13—B17—B20—B19	0.5 (9)
B9—B4—B5—B6	−35.3 (6)	B18—B17—B20—B19	−37.9 (7)
B8—B4—B5—B6	3.5 (9)	B12—B17—B20—B19	64.3 (8)
B3—B4—B5—B6	65.5 (8)	C5—B17—B20—B18	135.7 (7)
B1—B4—B5—B6	102.6 (7)	B13—B17—B20—B18	38.4 (7)
B8—B4—B5—B9	38.9 (6)	B12—B17—B20—B18	102.2 (7)
B3—B4—B5—B9	100.8 (7)	B15—B19—B20—C5	−1.6 (10)
B1—B4—B5—B9	137.9 (7)	B16—B19—B20—C5	−39.5 (6)
B9—B4—B5—B1	−137.9 (7)	B14—B19—B20—C5	61.6 (8)
B8—B4—B5—B1	−99.1 (7)	B18—B19—B20—C5	97.9 (7)
B3—B4—B5—B1	−37.1 (6)	B15—B19—B20—B16	37.8 (7)
B2—B5—B6—C2	38.3 (6)	B14—B19—B20—B16	101.0 (8)
B9—B5—B6—C2	−98.5 (7)	B18—B19—B20—B16	137.3 (7)
B1—B5—B6—C2	0.4 (9)	B15—B19—B20—B17	−62.7 (9)
B4—B5—B6—C2	−63.4 (8)	B16—B19—B20—B17	−100.5 (7)
B2—B5—B6—B9	136.8 (7)	B14—B19—B20—B17	0.5 (9)
B1—B5—B6—B9	98.9 (8)	B18—B19—B20—B17	36.8 (6)
B4—B5—B6—B9	35.1 (6)	B15—B19—B20—B18	−99.5 (8)
B2—B5—B6—B10	99.4 (7)	B16—B19—B20—B18	−137.3 (7)
B9—B5—B6—B10	−37.4 (6)	B14—B19—B20—B18	−36.3 (6)
B1—B5—B6—B10	61.5 (9)	B17—B18—B20—C5	37.8 (6)
B4—B5—B6—B10	−2.3 (9)	B14—B18—B20—C5	−63.1 (8)
B9—B5—B6—B2	−136.8 (7)	B13—B18—B20—C5	1.0 (9)
B1—B5—B6—B2	−37.9 (6)	B19—B18—B20—C5	−99.8 (7)
B4—B5—B6—B2	−101.7 (7)	B17—B18—B20—B16	100.0 (7)
C1—B2—B6—C2	−36.4 (5)	B14—B18—B20—B16	−0.9 (9)
B5—B2—B6—C2	−135.9 (7)	B13—B18—B20—B16	63.2 (9)
B1—B2—B6—C2	−97.4 (6)	B19—B18—B20—B16	−37.6 (6)
C1—B2—B6—B9	60.3 (8)	B14—B18—B20—B17	−100.9 (7)
C2—B2—B6—B9	96.7 (7)	B13—B18—B20—B17	−36.8 (7)
B5—B2—B6—B9	−39.2 (7)	B19—B18—B20—B17	−137.6 (7)
B1—B2—B6—B9	−0.7 (8)	B17—B18—B20—B19	137.6 (7)
C1—B2—B6—B5	99.5 (7)	B14—B18—B20—B19	36.8 (7)
C2—B2—B6—B5	135.9 (7)	B13—B18—B20—B19	100.9 (7)
B1—B2—B6—B5	38.5 (6)	B2—B1—C1—C2	−38.4 (6)
C1—B2—B6—B10	−2.2 (8)	B3—B1—C1—C2	103.2 (6)
C2—B2—B6—B10	34.2 (6)	B5—B1—C1—C2	0.4 (8)
B5—B2—B6—B10	−101.7 (8)	B4—B1—C1—C2	62.6 (7)
B1—B2—B6—B10	−63.3 (8)	B2—B1—C1—B7	−105.0 (7)
B8—B3—B7—C1	134.4 (7)	B3—B1—C1—B7	36.6 (7)
B1—B3—B7—C1	32.6 (6)	B5—B1—C1—B7	−66.2 (8)
B4—B3—B7—C1	96.0 (7)	B4—B1—C1—B7	−4.0 (9)
C1—B3—B7—C2	−36.6 (5)	B3—B1—C1—B2	141.6 (6)
B8—B3—B7—C2	97.8 (7)	B5—B1—C1—B2	38.8 (6)
B1—B3—B7—C2	−4.0 (8)	B4—B1—C1—B2	101.0 (6)
B4—B3—B7—C2	59.3 (7)	B2—B1—C1—B3	−141.6 (6)
C1—B3—B7—B8	−134.4 (7)	B5—B1—C1—B3	−102.8 (7)
B1—B3—B7—B8	−101.8 (7)	B4—B1—C1—B3	−40.6 (6)
B4—B3—B7—B8	−38.4 (6)	B2—B1—C1—Se1	113.0 (6)
C1—B3—B7—B10	−97.7 (7)	B3—B1—C1—Se1	−105.4 (6)
B8—B3—B7—B10	36.7 (6)	B5—B1—C1—Se1	151.8 (5)
B1—B3—B7—B10	−65.2 (7)	B4—B1—C1—Se1	−146.0 (5)
B4—B3—B7—B10	−1.8 (8)	B8—B7—C1—C2	−98.7 (7)
C1—B3—B8—B7	38.9 (6)	B3—B7—C1—C2	−138.4 (6)
B1—B3—B8—B7	98.9 (7)	B10—B7—C1—C2	−35.7 (6)
B4—B3—B8—B7	136.4 (7)	C2—B7—C1—B1	101.2 (7)
C1—B3—B8—B4	−97.5 (6)	B8—B7—C1—B1	2.6 (9)
B7—B3—B8—B4	−136.4 (7)	B3—B7—C1—B1	−37.2 (7)
B1—B3—B8—B4	−37.6 (6)	B10—B7—C1—B1	65.5 (8)
C1—B3—B8—B10	2.2 (8)	C2—B7—C1—B2	31.5 (6)
B7—B3—B8—B10	−36.7 (6)	B8—B7—C1—B2	−67.2 (8)
B1—B3—B8—B10	62.1 (8)	B3—B7—C1—B2	−107.0 (6)
B4—B3—B8—B10	99.7 (7)	B10—B7—C1—B2	−4.3 (8)
C1—B3—B8—B9	−60.2 (7)	C2—B7—C1—B3	138.4 (6)
B7—B3—B8—B9	−99.1 (7)	B8—B7—C1—B3	39.8 (6)
B1—B3—B8—B9	−0.2 (8)	B10—B7—C1—B3	102.7 (7)
B4—B3—B8—B9	37.3 (6)	C2—B7—C1—Se1	−112.0 (5)
C1—B7—B8—B3	−39.7 (6)	B8—B7—C1—Se1	149.4 (5)
C2—B7—B8—B3	−101.3 (6)	B3—B7—C1—Se1	109.6 (6)
B10—B7—B8—B3	−139.4 (7)	B10—B7—C1—Se1	−147.7 (5)
C1—B7—B8—B4	0.1 (9)	B5—B2—C1—C2	97.6 (6)
C2—B7—B8—B4	−61.5 (8)	B1—B2—C1—C2	138.3 (6)
B3—B7—B8—B4	39.8 (6)	B6—B2—C1—C2	35.6 (5)
B10—B7—B8—B4	−99.6 (7)	C2—B2—C1—B1	−138.3 (6)
C1—B7—B8—B10	99.7 (7)	B5—B2—C1—B1	−40.7 (6)
C2—B7—B8—B10	38.1 (6)	B6—B2—C1—B1	−102.7 (7)
B3—B7—B8—B10	139.4 (7)	C2—B2—C1—B7	−31.5 (6)
C1—B7—B8—B9	62.6 (8)	B5—B2—C1—B7	66.1 (8)
C2—B7—B8—B9	1.0 (8)	B1—B2—C1—B7	106.8 (7)
B3—B7—B8—B9	102.2 (7)	B6—B2—C1—B7	4.1 (8)
B10—B7—B8—B9	−37.2 (6)	C2—B2—C1—B3	−100.5 (6)
B9—B4—B8—B3	138.0 (7)	B5—B2—C1—B3	−2.9 (8)
B5—B4—B8—B3	99.5 (7)	B1—B2—C1—B3	37.9 (6)
B1—B4—B8—B3	36.8 (6)	B6—B2—C1—B3	−64.8 (7)
B9—B4—B8—B7	98.8 (7)	C2—B2—C1—Se1	107.1 (6)
B3—B4—B8—B7	−39.3 (6)	B5—B2—C1—Se1	−155.3 (5)
B5—B4—B8—B7	60.3 (9)	B1—B2—C1—Se1	−114.6 (6)
B1—B4—B8—B7	−2.4 (9)	B6—B2—C1—Se1	142.7 (5)
B9—B4—B8—B10	35.1 (6)	B8—B3—C1—C2	−1.4 (8)
B3—B4—B8—B10	−102.9 (7)	B7—B3—C1—C2	38.3 (6)
B5—B4—B8—B10	−3.4 (9)	B1—B3—C1—C2	−103.9 (6)
B1—B4—B8—B10	−66.1 (8)	B4—B3—C1—C2	−63.2 (7)
B3—B4—B8—B9	−138.0 (7)	B8—B3—C1—B1	102.5 (7)
B5—B4—B8—B9	−38.5 (6)	B7—B3—C1—B1	142.2 (7)
B1—B4—B8—B9	−101.2 (7)	B4—B3—C1—B1	40.7 (6)
C2—B6—B9—B10	−38.3 (6)	B8—B3—C1—B7	−39.6 (6)
B5—B6—B9—B10	−138.0 (7)	B1—B3—C1—B7	−142.2 (7)
B2—B6—B9—B10	−100.0 (7)	B4—B3—C1—B7	−101.4 (7)
C2—B6—B9—B4	63.1 (8)	B8—B3—C1—B2	64.9 (7)
B5—B6—B9—B4	−36.7 (7)	B7—B3—C1—B2	104.6 (6)
B10—B6—B9—B4	101.3 (7)	B1—B3—C1—B2	−37.6 (6)
B2—B6—B9—B4	1.3 (9)	B4—B3—C1—B2	3.1 (8)
C2—B6—B9—B5	99.8 (6)	B8—B3—C1—Se1	−140.8 (5)
B10—B6—B9—B5	138.0 (7)	B7—B3—C1—Se1	−101.1 (6)
B2—B6—B9—B5	38.0 (6)	B1—B3—C1—Se1	116.7 (6)
C2—B6—B9—B8	−0.6 (8)	B4—B3—C1—Se1	157.4 (5)
B5—B6—B9—B8	−100.4 (7)	B9—B6—C2—C3	152.4 (7)
B10—B6—B9—B8	37.7 (6)	B5—B6—C2—C3	−143.7 (7)
B2—B6—B9—B8	−62.3 (8)	B10—B6—C2—C3	113.6 (8)
B8—B4—B9—B10	−36.1 (6)	B2—B6—C2—C3	−105.5 (7)
B3—B4—B9—B10	0.8 (9)	B9—B6—C2—B10	38.8 (6)
B5—B4—B9—B10	101.0 (7)	B5—B6—C2—B10	102.7 (7)
B1—B4—B9—B10	63.7 (8)	B2—B6—C2—B10	140.9 (7)
B8—B4—B9—B6	−101.2 (7)	B9—B6—C2—C1	−64.1 (7)
B3—B4—B9—B6	−64.3 (9)	B5—B6—C2—C1	−0.2 (8)
B5—B4—B9—B6	35.8 (6)	B10—B6—C2—C1	−102.9 (6)
B1—B4—B9—B6	−1.4 (9)	B2—B6—C2—C1	38.1 (6)
B8—B4—B9—B5	−137.0 (6)	B9—B6—C2—B2	−102.1 (7)
B3—B4—B9—B5	−100.2 (7)	B5—B6—C2—B2	−38.2 (6)
B1—B4—B9—B5	−37.3 (6)	B10—B6—C2—B2	−140.9 (7)
B3—B4—B9—B8	36.9 (6)	B9—B6—C2—B7	1.3 (8)
B5—B4—B9—B8	137.0 (6)	B5—B6—C2—B7	65.2 (8)
B1—B4—B9—B8	99.7 (7)	B10—B6—C2—B7	−37.5 (6)
B2—B5—B9—B10	−1.1 (9)	B2—B6—C2—B7	103.4 (6)
B6—B5—B9—B10	38.1 (6)	B9—B10—C2—C3	−149.8 (7)
B1—B5—B9—B10	−64.4 (8)	B7—B10—C2—C3	107.6 (8)
B4—B5—B9—B10	−101.9 (7)	B8—B10—C2—C3	146.5 (7)
B2—B5—B9—B6	−39.2 (6)	B6—B10—C2—C3	−111.0 (8)
B1—B5—B9—B6	−102.5 (7)	B9—B10—C2—B6	−38.8 (6)
B4—B5—B9—B6	−140.0 (7)	B7—B10—C2—B6	−141.5 (7)
B2—B5—B9—B4	100.8 (7)	B8—B10—C2—B6	−102.5 (7)
B6—B5—B9—B4	140.0 (7)	B9—B10—C2—C1	65.0 (7)
B1—B5—B9—B4	37.5 (6)	B7—B10—C2—C1	−37.7 (6)
B2—B5—B9—B8	62.5 (8)	B8—B10—C2—C1	1.3 (8)
B6—B5—B9—B8	101.7 (7)	B6—B10—C2—C1	103.8 (6)
B1—B5—B9—B8	−0.8 (8)	B9—B10—C2—B2	−0.4 (8)
B4—B5—B9—B8	−38.3 (6)	B7—B10—C2—B2	−103.1 (7)
B3—B8—B9—B10	101.6 (7)	B8—B10—C2—B2	−64.1 (7)
B7—B8—B9—B10	37.9 (6)	B6—B10—C2—B2	38.4 (6)
B4—B8—B9—B10	140.0 (7)	B9—B10—C2—B7	102.7 (7)
B3—B8—B9—B6	63.5 (8)	B8—B10—C2—B7	39.0 (6)
B7—B8—B9—B6	−0.2 (9)	B6—B10—C2—B7	141.5 (7)
B4—B8—B9—B6	101.8 (8)	B1—C1—C2—C3	144.5 (7)
B10—B8—B9—B6	−38.1 (6)	B7—C1—C2—C3	−108.1 (7)
B3—B8—B9—B4	−38.3 (6)	B2—C1—C2—C3	105.1 (7)
B7—B8—B9—B4	−102.1 (7)	B3—C1—C2—C3	−146.9 (6)
B10—B8—B9—B4	−140.0 (7)	Se1—C1—C2—C3	−8.6 (9)
B3—B8—B9—B5	0.6 (8)	B1—C1—C2—B6	−0.2 (8)
B7—B8—B9—B5	−63.1 (8)	B7—C1—C2—B6	107.3 (7)
B4—B8—B9—B5	39.0 (6)	B2—C1—C2—B6	−39.5 (6)
B10—B8—B9—B5	−101.0 (7)	B3—C1—C2—B6	68.5 (7)
B6—B9—B10—C2	38.2 (6)	Se1—C1—C2—B6	−153.2 (5)
B4—B9—B10—C2	−64.1 (8)	B1—C1—C2—B10	−68.6 (7)
B5—B9—B10—C2	0.9 (9)	B7—C1—C2—B10	38.8 (6)
B8—B9—B10—C2	−100.0 (7)	B2—C1—C2—B10	−107.9 (6)
B6—B9—B10—B7	100.3 (7)	B3—C1—C2—B10	0.1 (8)
B4—B9—B10—B7	−1.9 (9)	Se1—C1—C2—B10	138.4 (5)
B5—B9—B10—B7	63.1 (8)	B1—C1—C2—B2	39.3 (6)
B8—B9—B10—B7	−37.8 (6)	B7—C1—C2—B2	146.7 (6)
B6—B9—B10—B8	138.2 (7)	B3—C1—C2—B2	107.9 (6)
B4—B9—B10—B8	35.9 (6)	Se1—C1—C2—B2	−113.7 (5)
B5—B9—B10—B8	100.9 (7)	B1—C1—C2—B7	−107.4 (6)
B4—B9—B10—B6	−102.2 (8)	B2—C1—C2—B7	−146.7 (6)
B5—B9—B10—B6	−37.2 (6)	B3—C1—C2—B7	−38.8 (6)
B8—B9—B10—B6	−138.2 (7)	Se1—C1—C2—B7	99.5 (6)
C1—B7—B10—C2	36.3 (6)	C1—B2—C2—C3	−108.8 (7)
B8—B7—B10—C2	135.2 (6)	B5—B2—C2—C3	151.2 (7)
B3—B7—B10—C2	98.9 (6)	B1—B2—C2—C3	−144.8 (7)
C1—B7—B10—B9	−60.2 (8)	B6—B2—C2—C3	112.9 (8)
C2—B7—B10—B9	−96.6 (7)	C1—B2—C2—B6	138.3 (6)
B8—B7—B10—B9	38.7 (6)	B5—B2—C2—B6	38.2 (6)
B3—B7—B10—B9	2.3 (8)	B1—B2—C2—B6	102.3 (7)
C1—B7—B10—B8	−98.9 (6)	C1—B2—C2—B10	99.8 (6)
C2—B7—B10—B8	−135.2 (6)	B5—B2—C2—B10	−0.3 (8)
B3—B7—B10—B8	−36.4 (6)	B1—B2—C2—B10	63.8 (7)
C1—B7—B10—B6	2.6 (9)	B6—B2—C2—B10	−38.5 (6)
C2—B7—B10—B6	−33.7 (6)	B5—B2—C2—C1	−100.0 (7)
B8—B7—B10—B6	101.5 (7)	B1—B2—C2—C1	−36.0 (5)
B3—B7—B10—B6	65.1 (8)	B6—B2—C2—C1	−138.3 (6)
B3—B8—B10—C2	−2.2 (8)	C1—B2—C2—B7	30.8 (5)
B7—B8—B10—C2	−39.0 (5)	B5—B2—C2—B7	−69.2 (8)
B4—B8—B10—C2	63.0 (8)	B1—B2—C2—B7	−5.2 (8)
B9—B8—B10—C2	98.3 (7)	B6—B2—C2—B7	−107.5 (7)
B3—B8—B10—B9	−100.5 (7)	C1—B7—C2—C3	107.6 (7)
B7—B8—B10—B9	−137.3 (7)	B8—B7—C2—C3	−153.3 (7)
B4—B8—B10—B9	−35.3 (6)	B3—B7—C2—C3	144.3 (7)
B3—B8—B10—B7	36.8 (6)	B10—B7—C2—C3	−113.9 (8)
B4—B8—B10—B7	102.0 (7)	C1—B7—C2—B6	−100.6 (6)
B9—B8—B10—B7	137.3 (7)	B8—B7—C2—B6	−1.5 (8)
B3—B8—B10—B6	−63.2 (8)	B3—B7—C2—B6	−63.8 (7)
B7—B8—B10—B6	−100.0 (7)	B10—B7—C2—B6	37.9 (6)
B4—B8—B10—B6	2.0 (8)	C1—B7—C2—B10	−138.4 (6)
B9—B8—B10—B6	37.3 (6)	B8—B7—C2—B10	−39.3 (6)
B9—B6—B10—C2	−135.2 (7)	B3—B7—C2—B10	−101.7 (7)
B5—B6—B10—C2	−96.9 (7)	B8—B7—C2—C1	99.1 (6)
B2—B6—B10—C2	−34.5 (6)	B3—B7—C2—C1	36.7 (5)
C2—B6—B10—B9	135.2 (7)	B10—B7—C2—C1	138.4 (6)
B5—B6—B10—B9	38.3 (6)	C1—B7—C2—B2	−30.9 (6)
B2—B6—B10—B9	100.7 (7)	B8—B7—C2—B2	68.3 (7)
C2—B6—B10—B7	34.3 (6)	B3—B7—C2—B2	5.9 (8)
B9—B6—B10—B7	−100.9 (8)	B10—B7—C2—B2	107.6 (7)
B5—B6—B10—B7	−62.6 (8)	B19—B16—C4—C5	−99.1 (7)
B2—B6—B10—B7	−0.2 (9)	B15—B16—C4—C5	−137.6 (6)
C2—B6—B10—B8	97.1 (7)	B20—B16—C4—C5	−36.0 (6)
B9—B6—B10—B8	−38.1 (6)	C5—B16—C4—B15	137.6 (6)
B5—B6—B10—B8	0.2 (9)	B19—B16—C4—B15	38.5 (6)
B2—B6—B10—B8	62.6 (8)	B20—B16—C4—B15	101.6 (7)
B14—B11—B12—C4	−96.0 (7)	C5—B16—C4—B11	100.4 (7)
B13—B11—B12—C4	−134.9 (7)	B19—B16—C4—B11	1.3 (8)
B15—B11—B12—C4	−33.3 (6)	B15—B16—C4—B11	−37.2 (7)
C4—B11—B12—C5	36.5 (5)	B20—B16—C4—B11	64.4 (8)
B14—B11—B12—C5	−59.5 (8)	C5—B16—C4—B12	30.8 (6)
B13—B11—B12—C5	−98.5 (6)	B19—B16—C4—B12	−68.3 (8)
B15—B11—B12—C5	3.2 (8)	B15—B16—C4—B12	−106.8 (7)
C4—B11—B12—B13	134.9 (7)	B20—B16—C4—B12	−5.2 (8)
B14—B11—B12—B13	39.0 (7)	C5—B16—C4—Se3	−111.0 (5)
B15—B11—B12—B13	101.7 (7)	B19—B16—C4—Se3	149.9 (5)
C4—B11—B12—B17	97.4 (7)	B15—B16—C4—Se3	111.4 (6)
B14—B11—B12—B17	1.4 (9)	B20—B16—C4—Se3	−147.0 (5)
B13—B11—B12—B17	−37.6 (6)	B19—B15—C4—C5	−0.5 (8)
B15—B11—B12—B17	64.1 (8)	B14—B15—C4—C5	−64.5 (8)
C4—B12—B13—B17	−98.8 (7)	B16—B15—C4—C5	39.1 (6)
C5—B12—B13—B17	−37.6 (6)	B11—B15—C4—C5	−103.2 (6)
B11—B12—B13—B17	−137.3 (7)	B19—B15—C4—B16	−39.6 (6)
C4—B12—B13—B11	38.5 (6)	B14—B15—C4—B16	−103.6 (7)
C5—B12—B13—B11	99.7 (6)	B11—B15—C4—B16	−142.4 (7)
B17—B12—B13—B11	137.3 (7)	B19—B15—C4—B11	102.7 (8)
C4—B12—B13—B18	−61.6 (8)	B14—B15—C4—B11	38.8 (6)
C5—B12—B13—B18	−0.4 (8)	B16—B15—C4—B11	142.4 (7)
B17—B12—B13—B18	37.3 (7)	B19—B15—C4—B12	65.8 (8)
B11—B12—B13—B18	−100.0 (7)	B14—B15—C4—B12	1.8 (9)
C4—B12—B13—B14	0.9 (8)	B16—B15—C4—B12	105.4 (7)
C5—B12—B13—B14	62.1 (8)	B11—B15—C4—B12	−36.9 (7)
B17—B12—B13—B14	99.8 (8)	B19—B15—C4—Se3	−139.9 (6)
B11—B12—B13—B14	−37.6 (7)	B14—B15—C4—Se3	156.1 (6)
C4—B11—B13—B17	−0.4 (9)	B16—B15—C4—Se3	−100.2 (6)
B14—B11—B13—B17	−98.7 (8)	B11—B15—C4—Se3	117.4 (6)
B12—B11—B13—B17	38.6 (6)	B14—B11—C4—C5	64.9 (7)
B15—B11—B13—B17	−61.5 (8)	B13—B11—C4—C5	0.8 (8)
C4—B11—B13—B12	−39.0 (6)	B12—B11—C4—C5	−38.4 (6)
B14—B11—B13—B12	−137.3 (7)	B15—B11—C4—C5	103.7 (7)
B15—B11—B13—B12	−100.1 (7)	B14—B11—C4—B16	−1.9 (9)
C4—B11—B13—B18	62.5 (8)	B13—B11—C4—B16	−66.0 (8)
B14—B11—B13—B18	−35.8 (6)	B12—B11—C4—B16	−105.2 (7)
B12—B11—B13—B18	101.6 (7)	B15—B11—C4—B16	36.9 (7)
B15—B11—B13—B18	1.5 (9)	B14—B11—C4—B15	−38.8 (6)
C4—B11—B13—B14	98.3 (7)	B13—B11—C4—B15	−102.9 (7)
B12—B11—B13—B14	137.3 (7)	B12—B11—C4—B15	−142.1 (7)
B15—B11—B13—B14	37.2 (6)	B14—B11—C4—B12	103.3 (7)
C4—B11—B14—B15	38.4 (6)	B13—B11—C4—B12	39.2 (6)
B13—B11—B14—B15	138.2 (7)	B15—B11—C4—B12	142.1 (7)
B12—B11—B14—B15	100.2 (7)	B14—B11—C4—Se3	−145.5 (6)
C4—B11—B14—B18	−63.5 (8)	B13—B11—C4—Se3	150.5 (5)
B13—B11—B14—B18	36.3 (7)	B12—B11—C4—Se3	111.3 (7)
B12—B11—B14—B18	−1.7 (9)	B15—B11—C4—Se3	−106.6 (7)
B15—B11—B14—B18	−101.8 (7)	B13—B12—C4—C5	99.0 (6)
C4—B11—B14—B19	1.7 (9)	B17—B12—C4—C5	37.4 (5)
B13—B11—B14—B19	101.5 (7)	B11—B12—C4—C5	138.1 (6)
B12—B11—B14—B19	63.5 (9)	C5—B12—C4—B16	−30.9 (6)
B15—B11—B14—B19	−36.7 (6)	B13—B12—C4—B16	68.1 (8)
C4—B11—B14—B13	−99.8 (7)	B17—B12—C4—B16	6.5 (8)
B12—B11—B14—B13	−38.0 (7)	B11—B12—C4—B16	107.2 (7)
B15—B11—B14—B13	−138.2 (7)	C5—B12—C4—B15	−100.8 (7)
B17—B13—B14—B11	102.2 (7)	B13—B12—C4—B15	−1.8 (8)
B12—B13—B14—B11	38.4 (6)	B17—B12—C4—B15	−63.4 (8)
B18—B13—B14—B11	139.4 (7)	B11—B12—C4—B15	37.3 (7)
B17—B13—B14—B15	63.9 (9)	C5—B12—C4—B11	−138.1 (6)
B12—B13—B14—B15	0.1 (10)	B13—B12—C4—B11	−39.1 (6)
B11—B13—B14—B15	−38.3 (7)	B17—B12—C4—B11	−100.8 (6)
B18—B13—B14—B15	101.1 (8)	C5—B12—C4—Se3	106.1 (6)
B17—B13—B14—B18	−37.2 (7)	B13—B12—C4—Se3	−154.9 (5)
B12—B13—B14—B18	−101.0 (7)	B17—B12—C4—Se3	143.4 (5)
B11—B13—B14—B18	−139.4 (7)	B11—B12—C4—Se3	−115.8 (6)
B17—B13—B14—B19	1.7 (9)	B16—B20—C5—C6	106.6 (8)
B12—B13—B14—B19	−62.1 (8)	B17—B20—C5—C6	−113.5 (8)
B11—B13—B14—B19	−100.5 (7)	B19—B20—C5—C6	145.8 (7)
B18—B13—B14—B19	38.9 (7)	B18—B20—C5—C6	−151.2 (7)
B11—B14—B15—C4	−38.3 (6)	B16—B20—C5—C4	−37.9 (6)
B18—B14—B15—C4	62.6 (8)	B17—B20—C5—C4	102.0 (6)
B19—B14—B15—C4	100.0 (7)	B19—B20—C5—C4	1.3 (8)
B13—B14—B15—C4	−1.1 (9)	B18—B20—C5—C4	64.3 (7)
B11—B14—B15—B19	−138.4 (7)	B16—B20—C5—B17	−139.9 (7)
B18—B14—B15—B19	−37.4 (7)	B19—B20—C5—B17	−100.7 (7)
B13—B14—B15—B19	−101.1 (8)	B18—B20—C5—B17	−37.8 (6)
B11—B14—B15—B16	−99.4 (7)	B17—B20—C5—B16	139.9 (7)
B18—B14—B15—B16	1.6 (9)	B19—B20—C5—B16	39.2 (6)
B19—B14—B15—B16	39.0 (6)	B18—B20—C5—B16	102.2 (7)
B13—B14—B15—B16	−62.1 (9)	B16—B20—C5—B12	−103.6 (7)
B18—B14—B15—B11	101.0 (7)	B17—B20—C5—B12	36.4 (6)
B19—B14—B15—B11	138.4 (7)	B19—B20—C5—B12	−64.3 (8)
B13—B14—B15—B11	37.2 (7)	B18—B20—C5—B12	−1.4 (8)
B14—B11—B15—C4	135.0 (7)	B16—C4—C5—C6	−105.9 (7)
B13—B11—B15—C4	96.7 (7)	B15—C4—C5—C6	−145.8 (7)
B12—B11—B15—C4	33.6 (6)	B11—C4—C5—C6	145.7 (7)
C4—B11—B15—B19	−96.9 (7)	B12—C4—C5—C6	106.5 (7)
B14—B11—B15—B19	38.1 (7)	Se3—C4—C5—C6	−6.4 (8)
B13—B11—B15—B19	−0.2 (9)	B16—C4—C5—B20	39.3 (6)
B12—B11—B15—B19	−63.2 (8)	B15—C4—C5—B20	−0.6 (8)
C4—B11—B15—B14	−135.0 (7)	B11—C4—C5—B20	−69.0 (7)
B13—B11—B15—B14	−38.3 (6)	B12—C4—C5—B20	−108.2 (6)
B12—B11—B15—B14	−101.3 (7)	Se3—C4—C5—B20	138.9 (6)
C4—B11—B15—B16	−33.0 (6)	B16—C4—C5—B17	107.5 (6)
B14—B11—B15—B16	102.0 (7)	B15—C4—C5—B17	67.6 (7)
B13—B11—B15—B16	63.6 (8)	B11—C4—C5—B17	−0.9 (8)
B12—B11—B15—B16	0.6 (9)	B12—C4—C5—B17	−40.1 (6)
B19—B15—B16—C4	135.0 (7)	Se3—C4—C5—B17	−153.0 (5)
B14—B15—B16—C4	95.4 (7)	B15—C4—C5—B16	−39.9 (6)
B11—B15—B16—C4	32.9 (6)	B11—C4—C5—B16	−108.4 (7)
C4—B15—B16—C5	−37.2 (5)	B12—C4—C5—B16	−147.6 (6)
B19—B15—B16—C5	97.8 (7)	Se3—C4—C5—B16	99.5 (6)
B14—B15—B16—C5	58.3 (8)	B16—C4—C5—B12	147.6 (6)
B11—B15—B16—C5	−4.2 (8)	B15—C4—C5—B12	107.7 (7)
C4—B15—B16—B19	−135.0 (7)	B11—C4—C5—B12	39.2 (6)
B14—B15—B16—B19	−39.6 (7)	Se3—C4—C5—B12	−112.9 (6)
B11—B15—B16—B19	−102.0 (8)	B13—B17—C5—C6	−144.7 (7)
C4—B15—B16—B20	−97.6 (7)	B18—B17—C5—C6	151.1 (7)
B19—B15—B16—B20	37.4 (7)	B12—B17—C5—C6	−105.9 (8)
B14—B15—B16—B20	−2.1 (9)	B20—B17—C5—C6	111.6 (8)
B11—B15—B16—B20	−64.6 (8)	B13—B17—C5—B20	103.7 (7)
B12—B13—B17—C5	38.7 (6)	B18—B17—C5—B20	39.6 (6)
B11—B13—B17—C5	−0.1 (9)	B12—B17—C5—B20	142.5 (7)
B18—B13—B17—C5	−99.5 (7)	B13—B17—C5—C4	0.6 (8)
B14—B13—B17—C5	−62.4 (8)	B18—B17—C5—C4	−63.6 (7)
B12—B13—B17—B18	138.1 (7)	B12—B17—C5—C4	39.4 (5)
B11—B13—B17—B18	99.4 (7)	B20—B17—C5—C4	−103.1 (6)
B14—B13—B17—B18	37.1 (7)	B13—B17—C5—B16	64.9 (8)
B11—B13—B17—B12	−38.8 (6)	B18—B17—C5—B16	0.7 (8)
B18—B13—B17—B12	−138.1 (7)	B12—B17—C5—B16	103.7 (6)
B14—B13—B17—B12	−101.1 (8)	B20—B17—C5—B16	−38.8 (6)
B12—B13—B17—B20	99.7 (7)	B13—B17—C5—B12	−38.8 (6)
B11—B13—B17—B20	61.0 (9)	B18—B17—C5—B12	−102.9 (7)
B18—B13—B17—B20	−38.4 (7)	B20—B17—C5—B12	−142.5 (7)
B14—B13—B17—B20	−1.3 (9)	C4—B16—C5—C6	109.4 (7)
C4—B12—B17—C5	−37.7 (5)	B19—B16—C5—C6	−152.7 (7)
B13—B12—B17—C5	−136.1 (7)	B15—B16—C5—C6	146.3 (7)
B11—B12—B17—C5	−98.5 (7)	B20—B16—C5—C6	−112.8 (8)
C4—B12—B17—B13	98.4 (7)	C4—B16—C5—B20	−137.8 (6)
C5—B12—B17—B13	136.1 (7)	B19—B16—C5—B20	−39.9 (6)
B11—B12—B17—B13	37.6 (6)	B15—B16—C5—B20	−100.9 (7)
C4—B12—B17—B18	60.1 (8)	B19—B16—C5—C4	97.9 (7)
C5—B12—B17—B18	97.8 (7)	B15—B16—C5—C4	36.9 (6)
B13—B12—B17—B18	−38.3 (7)	B20—B16—C5—C4	137.8 (6)
B11—B12—B17—B18	−0.6 (9)	C4—B16—C5—B17	−98.5 (6)
C4—B12—B17—B20	−4.9 (8)	B19—B16—C5—B17	−0.6 (9)
C5—B12—B17—B20	32.8 (6)	B15—B16—C5—B17	−61.7 (8)
B13—B12—B17—B20	−103.3 (8)	B20—B16—C5—B17	39.3 (7)
B11—B12—B17—B20	−65.7 (8)	C4—B16—C5—B12	−30.3 (6)
C5—B17—B18—B14	62.5 (8)	B19—B16—C5—B12	67.6 (8)
B13—B17—B18—B14	−38.0 (7)	B15—B16—C5—B12	6.6 (8)
B12—B17—B18—B14	−0.4 (9)	B20—B16—C5—B12	107.5 (7)
B20—B17—B18—B14	100.3 (8)	C4—B12—C5—C6	−109.6 (7)
C5—B17—B18—B13	100.5 (7)	B13—B12—C5—C6	152.2 (7)
B12—B17—B18—B13	37.6 (7)	B17—B12—C5—C6	114.0 (7)
B20—B17—B18—B13	138.4 (7)	B11—B12—C5—C6	−145.8 (7)
C5—B17—B18—B20	−37.9 (6)	C4—B12—C5—B20	99.3 (6)
B13—B17—B18—B20	−138.4 (7)	B13—B12—C5—B20	1.1 (8)
B12—B17—B18—B20	−100.7 (7)	B17—B12—C5—B20	−37.1 (6)
C5—B17—B18—B19	−0.5 (8)	B11—B12—C5—B20	63.1 (8)
B13—B17—B18—B19	−101.0 (7)	B13—B12—C5—C4	−98.2 (6)
B12—B17—B18—B19	−63.3 (8)	B17—B12—C5—C4	−136.4 (6)
B20—B17—B18—B19	37.4 (7)	B11—B12—C5—C4	−36.2 (5)
B11—B14—B18—B17	1.3 (9)	C4—B12—C5—B17	136.4 (6)
B15—B14—B18—B17	−64.1 (9)	B13—B12—C5—B17	38.2 (6)
B19—B14—B18—B17	−100.2 (8)	B11—B12—C5—B17	100.2 (7)
B13—B14—B18—B17	37.3 (6)	C4—B12—C5—B16	30.0 (6)
B11—B14—B18—B13	−36.0 (7)	B13—B12—C5—B16	−68.2 (7)
B15—B14—B18—B13	−101.4 (7)	B17—B12—C5—B16	−106.4 (7)
B19—B14—B18—B13	−137.5 (7)	B11—B12—C5—B16	−6.2 (8)
B11—B14—B18—B20	64.9 (9)	C2—C1—Se1—Se2	106.1 (5)
B15—B14—B18—B20	−0.5 (9)	B1—C1—Se1—Se2	−43.2 (6)
B19—B14—B18—B20	−36.6 (6)	B7—C1—Se1—Se2	173.6 (4)
B13—B14—B18—B20	100.9 (7)	B2—C1—Se1—Se2	34.2 (6)
B11—B14—B18—B19	101.5 (7)	B3—C1—Se1—Se2	−117.9 (4)
B15—B14—B18—B19	36.1 (6)	C1—Se1—Se2—Se3	−95.3 (2)
B13—B14—B18—B19	137.5 (7)	C5—C4—Se3—Se2	104.6 (5)
B12—B13—B18—B17	−37.7 (6)	B16—C4—Se3—Se2	171.9 (4)
B11—B13—B18—B17	−102.1 (7)	B15—C4—Se3—Se2	−118.8 (5)
B14—B13—B18—B17	−137.8 (7)	B11—C4—Se3—Se2	−43.1 (6)
B17—B13—B18—B14	137.8 (7)	B12—C4—Se3—Se2	33.7 (6)
B12—B13—B18—B14	100.0 (7)	Se1—Se2—Se3—C4	−84.8 (2)
